# Multivariate analysis of ecotypic responses of *Scrophularia striata* to germination-enhancing treatments

**DOI:** 10.1186/s12870-026-08440-x

**Published:** 2026-04-23

**Authors:** Tahereh Movahhed Haghighi, Seyyed Sasan Mousavi, Azin Taban, Hossein Sadeghi, Elham Esmailpourmoghadam

**Affiliations:** 1 Department of Agriculture, Iranian Research Organization for Science and Technology (IROST), Tehran, Iran; 2 Department of Plant Production, College of Agriculture and Natural Resources of Darab, Shiraz University, Shiraz, Iran; 3 Department of Horticultural Science, Faculty of Agriculture, Shiraz University, Shiraz, Iran; 4 Department of Natural Resources and Environmental Engineering, School of Agriculture, Shiraz University, Shiraz, Iran; 5 Department of Life Science and Technology, School of Life Science and Technology, Institute of Science Tokyo, Yokohama, Japan

**Keywords:** Gibberellic acid, Multivariate analysis, Seed dormancy, Seed enhancement techniques, UV-B radiation

## Abstract

This study aimed to develop seed-based priming techniques for the conservation and medicinal uses of *Scrophularia striata*. Seeds were collected from two ecotypes and subjected to untreated (control), hydropriming (H), hot water soaking (HW), gibberellic acid (GA), potassium nitrate (KN), salicylic acid (SA), and UV-B exposure treatments. A comprehensive assessment of germination and initial seedling growth parameters was conducted to compare the efficacy of applied treatments. Results showed that GA treatments markedly improved germination speed and uniformity, leading to an 80.8% increase in the coefficient of velocity of germination, compared to the control. Moderate GA concentrations (75 and 150 mg/L) also enhanced seedling growth by up to 90% and increased final germination by more than 50%, compared to the control. Hydropriming for 72 h effectively accelerated germination onset, shortening the first day of germination by 56.7% compared to the control. Multivariate analysis using metric multidimensional scaling (MDS) clearly distinguished treatment responses, with a very low stress value for the two ecotypes (0.0334 for Lizan and 0.0184 for Pahleh), indicating an excellent model fit. The MDS plots highlighted GA as the most effective priming method, while H showed moderate but consistent improvement, clustering near the control and indicating partial enhancement of germination traits. Conversely, KN and heat-based treatments were less effective. The two ecotypes differed in their responses: Lizan was more sensitive to GA concentration and H duration, whereas Pahleh exhibited more uniform enhancement across treatments. Therefore, the study outcomes will provide fundamental and practical insights into the efficiency of germination-enhancing methods across ecotypes and provide valuable insights for optimizing pre-sowing strategies in medicinal plant cultivation.

**Graphical Abstract:**

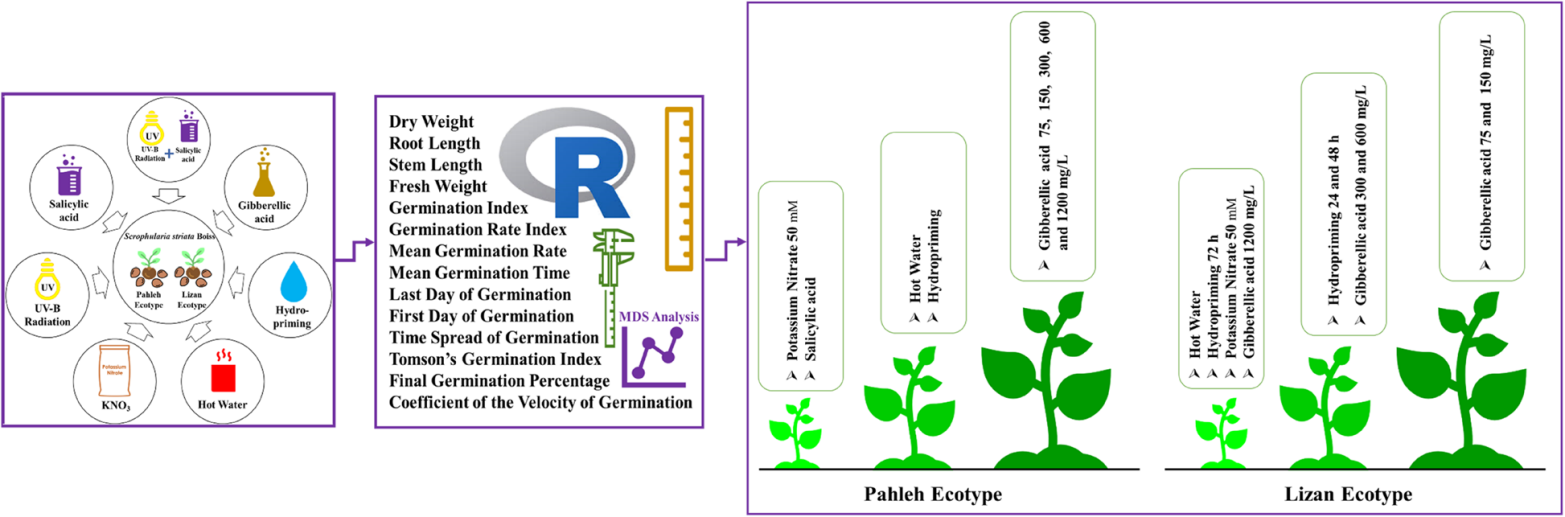

**Supplementary Information:**

The online version contains supplementary material available at 10.1186/s12870-026-08440-x.

## Introduction

Seed germination is a critical phase in the plant life cycle and strongly influences seedling establishment, population dynamics, and crop productivity [1]. In many species, germination is restricted by physiological or endogenous dormancy, which delays or prevents radicle emergence even under favorable environmental conditions [2]. Seed priming, a controlled pre-sowing physiological technique that partially hydrates seeds and activates early metabolic processes, without allowing radicle emergence, has been widely recognized as an effective strategy to overcome dormancy and improve germination performance [3]. Properly optimized priming treatments can accelerate germination, enhance uniformity, and promote early seedling vigor, thereby contributing to plant establishment and subsequent growth. Primed seeds resume cellular activities more rapidly due to reduced imbibition time, leading to synchronized radicle emergence, improved early growth, and better-developed root and shoot systems [4, 5]. These effects are associated with enhanced enzyme activity, more efficient mobilization of stored reserves, and improved cellular repair mechanisms during the early stages of germination [6]. Owing to its simplicity, effectiveness, low-cost, and minimal environmental risk technique, seed priming has been increasingly applied in both agricultural and ecological contexts as a practical means of improving plant performance [7, 8].

A wide range of physical, chemical, and hormonal priming methods has been developed to enhance seed germination [8]. Hydropriming (H), which involves soaking seeds in water for a defined period, is the most accessible and environmentally sustainable approach, with no specialized inputs. Osmopriming using compounds such as potassium nitrate (KN) has also been extensively applied; nitrate ions not only serve as a nutrient source but also function as a signaling molecule that stimulates germination-related metabolic pathways and helps alleviate seed dormancy. Hormonal priming with gibberellic acid (GA) plays a critical role in germination regulation by promoting the synthesis of hydrolytic enzymes that facilitate the mobilization of endosperm reserves required for embryo growth. Gibberellic acid may regulate plant height and shoot biomass in a balance with cytokinin and auxin [6]. However, the effects of GA are concentration-dependent, and excessive levels may negatively affect germination or subsequent seedling development [9]. Physical priming methods, including thermo-priming, hot water (HW) treatment, and radiation priming represents another effective method for improving seed germination, particularly for species exhibiting physical or physiological dormancy [10]. Exposure to elevated temperatures can soften or disrupt the seed coat structure, increase permeability, and enhance water uptake and gas exchange. Additionally, HW treatment may reduce surface-borne pathogens and inhibitory substances, indirectly contributing to improved germination [11]. Chemical priming with signaling molecules such as salicylic acid (SA) has also gained attention due to its role in regulating plant physiological responses and redox homeostasis [12]. During germination, SA priming may enhance antioxidant capacity, reduce oxidative damage, and improve seedling establishment, particularly under suboptimal conditions [13]. Moreover, ultraviolet (UV) radiation, as a physical priming agent, has been proposed to stimulate moderate oxidative signaling through the generation of reactive oxygen species (ROS), such as hydrogen peroxide (H_2_O_2_) and superoxide (O_2_^−^), which can activate stress-responsive pathways, occasionally resulting in beneficial physiological responses [14].

Plants ecotypes originating from different geographical and environmental conditions often exhibit distinct genetic backgrounds and physiological characteristics that influence dormancy mechanisms, germination behavior, and early seedling growth. As a result, the effectiveness of seed priming treatments is not universal and may vary substantially even among ecotypes. Ecotype-specific responses reflect differences in metabolic activity, hormone sensing, and adaptive strategies shaped by local environmental pressures [15]. Understanding the interaction between ecotype and priming treatment (Ecotype × Priming) and ecotype-specific responses to seed priming is essential for optimizing germination strategies and improving seedling establishment. It can provide valuable insights into adaptive traits and physiological plasticity. Despite its importance, comparative evaluation of ecotype-dependent responses to various priming methods remains limited, particularly in medicinal plant species.

*Scrophularia striata* (Scrophulariaceae) is a medicinal plant of considerable pharmacological interest, known for its anti-inflammatory, antioxidant, and antimicrobial effects. Successful propagation of *S. striata* is constrained by low and inconsistent germination due to endogenous dormancy. Previous studies have shown that treatments such as scarification (physical or mechanical), moist chilling, and soaking in GA can partially overcome dormancy and improve germination in this species [16, 17]. However, these studies have generally focused on individual treatments and have not addressed potential variability among ecotypes or the combined effects of different priming strategies.

Most existing research on seed priming has evaluated single methods in isolation and relied primarily on univariate statistical analyses. Such approaches may overlook complex, multidimensional relationships among germination traits and treatment responses, particularly when multiple priming methods and ecotypes are involved. Multivariate statistical techniques, including multidimensional scaling (MDS), clustering, and principal component analysis (PCA) provides powerful tools for simultaneously analyzing multiple traits and visualizing patterns of similarity or divergence among treatments and ecotypes [18]. These methods can reveal interaction effects and response syndromes that are not readily apparent through single-trait comparisons [19]. MDS is used for the visualization and exploratory analysis of multidimensional data. It aims to represent the structure of a set of objects based on their pairwise dissimilarities in a low-dimensional space, typically two or three dimensions [20]. MDS is used to discover structures within data by displaying variations among pairs of elements as distances between points in a low-dimensional space. MDS is broadly utilized for representing complex data sets in a more readable form and facilitates recognizing patterns and structures within the data [21, 22].

Therefore, the present study was designed to provide a comprehensive evaluation of physical, chemical, and combined seed priming strategies using a multivariate analytical framework to show new insights into *S. striata* seed germination. By using multivariate statistical analysis, this research presents a novel aspect of their effectiveness alone or integrated with other treatments cause a valuable perspective to break seed dormancy and improve germination rate. Therefore, we aim to systematically examine the effects of multiple seed priming methods, including H, GA, KN, SA, SA integrated UV-B exposure, and HW treatments, on germination and early seedling performance across different ecotypes of *S. striata*.

It was hypothesized that seed priming would significantly enhance germination performance and seedling vigor, compared to untreated seeds. Moreover, the magnitude and direction of these responses would differ among ecotypes due to inherent genetic and physiological variation. Moreover, multivariate analyses would reveal ecotype-specific response patterns and treatment groupings that cannot be fully resolved using single-trait comparisons. By identifying ecotype-dependent priming responses, this study aimed to provide a scientific basis for optimizing propagation strategies and improving the cultivation and utilization of *S. striata.* This study represents the first comprehensive multivariate comparison of physical and chemical priming strategies across distinct ecotypes of *S. striata.*

## Materials and methods

### Experimental site

*S. striata* is predominantly distributed in the province of Ilam, a mountainous region, in the western part of Iran [23]. To choose the sampling location, habitats of the species were identified through field surveys. Subsequently, regarding distinct ecological elements including temperature, soil, and latitude, two regions, namely Lizan (Longitude: 46° 8′ 19.42″ E; Latitude: 33° 34′ 2.16″ N) and Pahleh (Longitude: 46° 50′ 43.16″E; Latitude: 33° 2′ 27.28″ N), located approximately 149 km apart from each other, were chosen [17].

### Plant material

Seeds of *S. striata* were gathered from two locations in Ilam province: Lizan and Pahleh. The collection of samples was carried out under national and scientific guidelines as described by Esmaeili et al. (2020) [24], and was based on International Standards for Sustainable Wild Collection of Medicinal and Aromatic Plants (ISSC-MAP) (Version 1.0), according to the Medicinal Plant Specialist Group Species Survival Commission of IUCN (The World Conservation Union). The permission to collect plants from wild natural resources was obtained from the Iranian Organization of Forests and Rangeland (Government Organization). After collection, seeds were cleaned to remove debris and were air-dried before storing. Seeds from the two ecotypes were stored in a cool, dry, and dark environment with adequate ventilation. During collection, about 20 mother bushes were designated to represent the seed sources of the local populations [25, 26].

### Treatments

In this experiment, seeds from two different ecotypes were examined under various treatments to evaluate germination and initial growth parameters. The treatments included H for different durations of 24, 48, and 72 h at room temperature to assess the impact of water absorption on germination and initial growth indices. Additionally, gibberellic acid (GA) was applied at concentrations of 75, 150, 300, 600, and 1200 mg/L to examine its role in promoting seed germination. Potassium nitrate (KN) treatments were also applied at 50, 250, 450, 650, and 850 mM. Seeds were soaked in the respective solutions for 24 h with aeration. For each concentration, 90 seeds were used. Following treatment, seeds were removed from the solution and immediately transferred to Petri dishes without washing or drying. Furthermore, seeds were subjected to hot HW (80–85 °C) soaking treatments for 1, 3, and 5 min to assess the influence of temperature on germination potential. Seeds were taken out of the HW and immediately transferred to Petri dishes without drying. To explore the interactive effects of SA and UV-B radiation (20 W/m^2^), seeds were treated with SA at concentrations of 0.1, 0.25, and 0.5 mM both with and without UV-B exposure. The treatment was performed first by UV-B exposure, followed by soaking them in the SA solutions. The UV-B lamp was bought from the M.M.G.S. company, and the manufacturing factory was Philips, Germany (Voltage: 59 V; Power: 20 W; Current: 370 mA). The distance between the source and the sample was 20 cm, and the seeds were exposed to radiation for 30 min. Distilled water was used as a control treatment. All experiments were carried out using seeds from the two distinct ecotypes, Lizan and Pahleh.

### Germination assessment

The experiments were conducted in Petri dishes within a growth room under controlled light and temperature conditions. Whatman Grade 1 porous filter paper was located in Petri dishes, humidified with distilled water, and periodically rehydrated. For each treatment, seeds from both ecotypes of *S. striata* were used with three replicates consisting of 30 seeds per Petri dish, totaling 90 seeds per treatment. Seeds placed in Petri dishes were kept in a phytotron (1300 STC Mod, Noor-Sanat-Ferdows Company, Karaj, Iran) at 25 °C, with a light intensity of 4000 lx and a 16-h photoperiod. The dishes were checked daily. Germination was monitored for up to 10 days, and the number of germinated seeds was recorded. At the conclusion of the experiment, both germinated and ungerminated seeds were counted. A seed was considered germinated when the radicle had emerged to grow at least 2 mm [27, 28]. For each treatment, all germinated seedlings in a Petri dish were measured. Each Petri dish served as a single replicate. The mean of the seedlings within each dish was calculated and reported as the replicate value for that treatment. The data collected daily were used to compute ten different germination parameters, including final germination percentage (FGP) [29], mean germination time (MGT) (the mean time taken by the overall seeds to complete the mechanism of germination [30]), mean germination rate (MGR) [31], germination rate index (GRI) [29], germination index (GI), the first day of germination (FDG) (time for the first germination to occur), last day of germination (LDG) (time for the last germination to occur), coefficient of the velocity of germination (CVG), [32, 33], Timson’s germination index (TGI), and time spread of germination (TSG) (the difference between the time for last germination and time for first germination) [29, 31, 34–37]. Root and stem sizes were quantified by ruler, and seedlings’ fresh and dry weights were measured by a 4-digit scale [38]. All formulas and their explanations are shown in Supplementary Table 1.

### Statistical analysis

The study was conducted using a completely randomized design (CRD), with each treatment replicated three times. Statistical significance was evaluated using a two-way analysis of variance (ANOVA) followed by the Duncan test. All data were analyzed using IBM SPSS statistical software (v. 21), and mean comparisons were performed with Duncan’s test at a 5% significance level. The relationships between treatments and germination indices were examined through PCA, with Pearson’s correlation coefficient used to indicate associations. PCA was also conducted using IBM SPSS (v. 21). Clustering analysis was performed using RStudio (2024.12.0 Build 467) using the three packages, including factoextra, cluster, and tidyverse. Hierarchical clustering with Ward’s method was applied to scaled data based on Euclidean distance. PCA was performed to identify dominant patterns of variation and evaluate contributions of individual germination and seedling traits. MDS was applied as a complementary distance-based ordination method to visualize treatment-level similarities. Metric MDS was preferred over NMDS because the data were continuous, quantitative, and well-behaved, allowing the preservation of actual distances among treatments. The very low MDS stress value (0.0334) confirms an excellent fit of the ordination.

## Results

### Seed germination indices of *S. striata* ecotypes

**Fig. 1:**
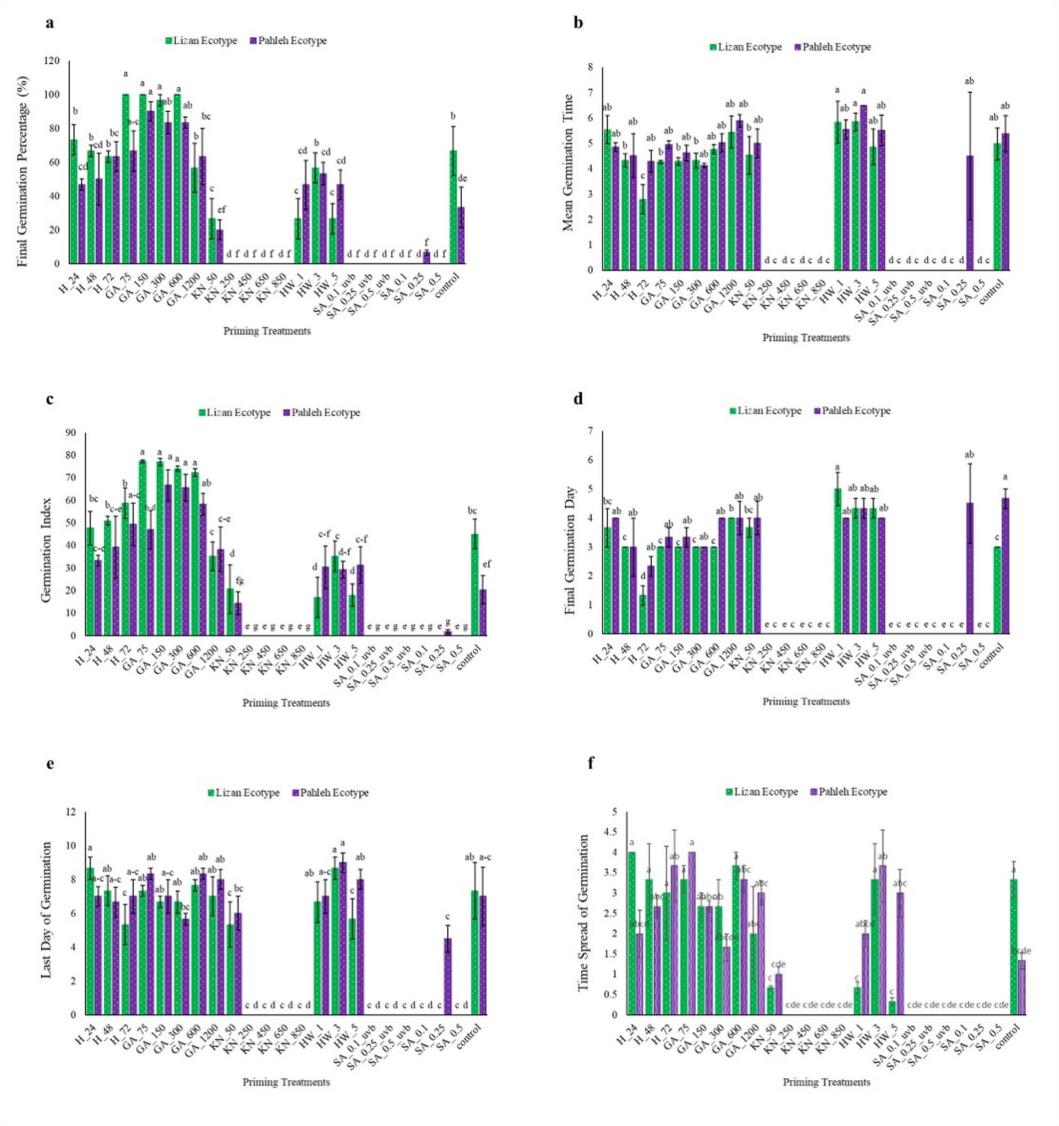
Germination parameters of *S. striata* ecotypes (Lizan and Pahleh) under the studied treatments (**a**) Final germination percentage (FGP), **b** Mean germination time (per day) (MGT), **c** Germination index (GI), and **d** First day of germination (FDG). **e** Last day of germination (LDG), and **f** Time spread of germination (TSG). 24, 48, and 72 h hydropriming (H); 75, 150, 300, 600, and 1200 mg/L gibberellin (GA); 50, 250, 450, 650 and 850 mM potassium nitrate (KN); 1-, 3- and 5-min soaking in hot water (HW) and 0.1, 0.25 and 0.5 mM salicylic acid (SA) with and without UV-B exposure. Statistical significance was evaluated using a two-way ANOVA followed by Duncan test. Groups denoted by identical letters did not differ significantly, whereas groups assigned different letters exhibited statistically significant differences at *P* < 0.05. Data are expressed as means ± SE (n = 3)

The FGP varied significantly among priming treatments and ecotypes (Fig. 1a). H for 24 h increased the FGP value by 10% in Lizan, and H for 72 h increased FGP by 90% in Pahleh, compared to the control. The Lizan ecotype exhibited the highest germination rates (> 95%) under GA treatments (75–600 mg/L), surpassing those of the Pahleh ecotype. In contrast, KN (50–850 mM) and SA (0.1–0.5 mM) treatments, regardless of UV-B exposure, yielded minimal effects on germination of both ecotypes (< 5%). HW soaking resulted in moderate germination, comparable to the control. The control treatment demonstrated significantly lower germination rates in both ecotypes than in GA treatments (Fig. 1a). Mean germination time was generally shorter in the Lizan ecotype than in the Pahleh across most priming treatments (Fig. 1b). GA and HW treatments showed MGT values statistically identical to those observed in the control. No germination was recorded under KN (250–850 mM) and SA + UV-B treatments This inhibition could be related to osmotic stress or hormonal imbalance, but this requires further physiological verification (Fig. 1b). GA treatments (75, 150, 300, and 600 mg/L) significantly increased the germination indices (GI) in both ecotypes, especially in Lizan (Fig. 1c), highlighting its effectiveness as a germination promoter. SA treatments (regardless of UV-B exposure), and KN treatments (250–850 mM) had a significantly adverse effect on the GI value germination index, suggesting inhibitory properties at these compounds (Fig. 1c). Interestingly, 72 h H significantly reduced the FDG of Lizan ecotype compared to the control, making it the only effective treatment in FDG that exhibited a statistically significant difference. No germination was observed under KN (250–850 mM) treatments. In contrast, the lowest KN concentration (50 mM) did not affect seed germination in both ecotypes, indicating the negative impact of KN, especially in high concentrations. Similarly, all SA treatments, except for 0.25 mM, resulted in no germination, suggesting a potential inhibitory effect of SA on seed germination. While 75–600 mg/L of GA treatments were comparable with the control treatment in these two ecotypes. To achieve faster germination (lower FDG), 72 h of H and low to moderate concentrations of GA appear to be promising strategies (Fig. 1d).

**Fig. 2:**
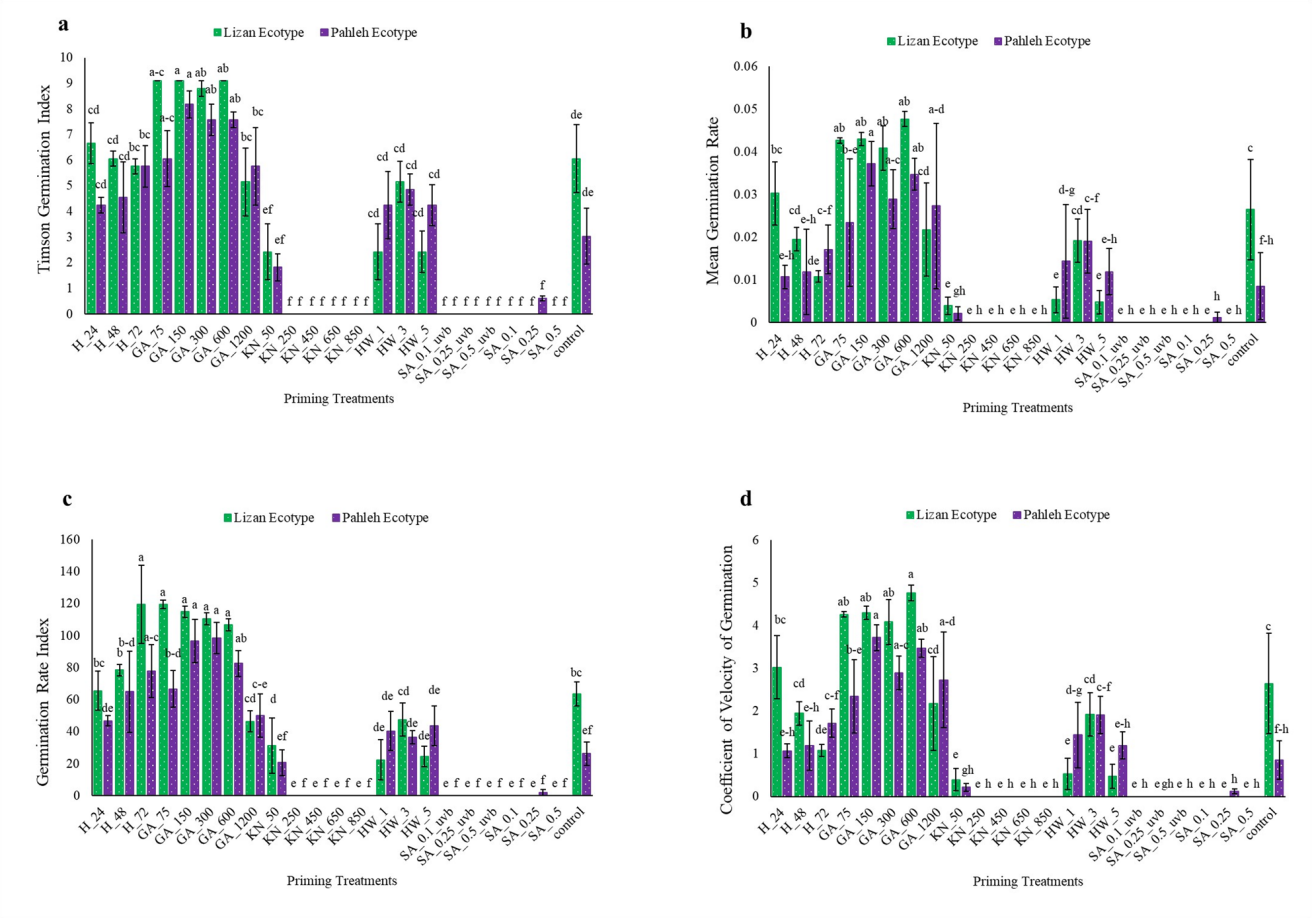
**a** Timson germination index (TGI), **b** Average germination rate (per day) (MGR), **c** Germination rate index (GRI), and **d** Coefficient of velocity of germination (CVG) of *S. striata* ecotypes under the studied treatments including: 24, 48, and 72 h hydropriming (H); 75, 150, 300, 600, and 1200 mg/L gibberellin (GA); 50, 250, 450, 650 and 850 mM potassium nitrate (KN); 1, 3 and 5 min soaking in hot water (HW) and 0.1, 0.25 and 0.5 mM salicylic acid (SA) with and without UV-B exposure. Statistical significance was evaluated using a two-way ANOVA followed by Duncan test. Groups denoted by identical letters did not differ significantly, whereas groups assigned different letters exhibited statistically significant differences at *P* < 0.05. Data are expressed as means ± SE (n = 3)

The TGI revealed significant variation among treatments, with GA (75–600 mg/L) demonstrating the highest germination performance. In contrast, SA + UV-B and KN treatments yielded markedly low germination indices, approaching zero. No significant differences were observed between HW treatments and the control (Fig. 2a). The Lizan ecotype exhibited the highest mean germination rate under GA treatments (75–600 mg/L) compared to the control. In contrast, HW resulted in lower germination rates than GA treatments, potentially due to excessive thermal exposure causing protein denaturation or cellular damage. The complete inhibition of germination observed in SA and KN treatments suggests possible ion toxicity or osmotic stress at the applied concentrations, which may have impeded water uptake or disrupted hormonal signaling pathways (Fig. 2b). Calculations of the germination rate index revealed that H for 72 h and most of GA concentrations (75–600 mg/L) accelerated and synchronized seed germination in both ecotypes compared with the control. In contrast, the other treatments exerted no positive effect on the germination rate index (Fig. 2c). The CVG data indicated that GA treatments ranging from 75 to 600 mg L were the most effective in accelerating seed germination and reducing potential dormancy constraints (Fig. 2d).

### Initial growth performance of *S. striata* ecotypes under the studied treatments

**Fig. 3:**
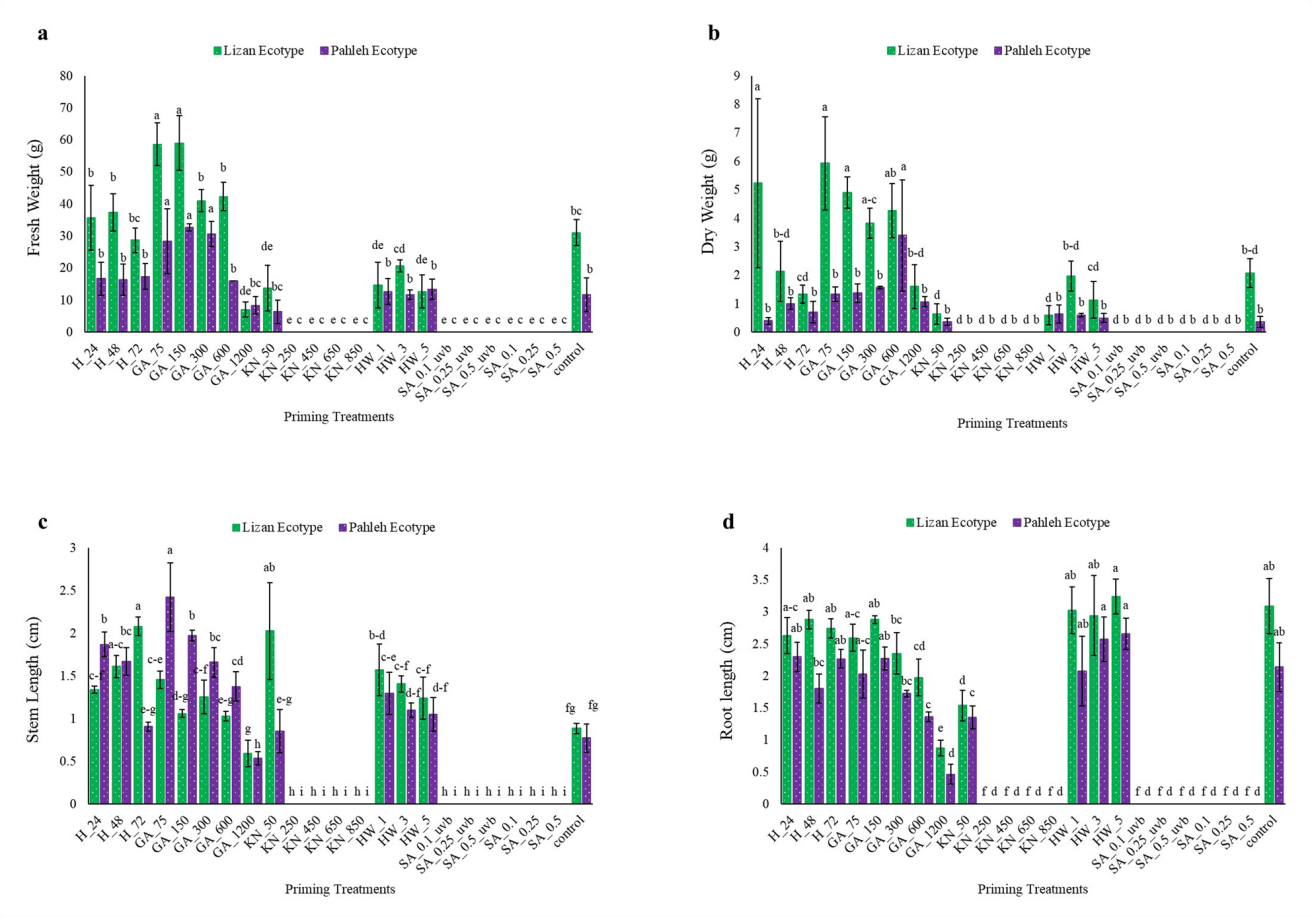
Seedling performance of *S. striata* ecotypes (Lizan and Pahleh) under the studied treatments (**a**) Fresh weight, **b** Dry weight, **c** Stem length, and **d** Root length. H_24, 48, and 72 (24, 48, and 72 h hydropriming), GA_75, 150, 300, 600, and 1200 [gibberellin (GA) 75, 150, 300, 600, and 1200 mg/L], KN_50, 250, 450, 650 and 850 [Potassium nitrate (KN) 50, 250, 450, 650 and 850 mM], HW_1, 3, and 5 (hot water soaking for 1, 3, and 5 min), and SA_0.1, 0.25, and 0.5/_UV-B (salicylic acid 0.1, 0.25 and 0.5 mM with and without UV-B exposure). Statistical significance was evaluated using a two-way ANOVA followed by Duncan test. Groups denoted by identical letters did not differ significantly, whereas groups assigned different letters exhibited statistically significant differences at *P* < 0.05. Data are expressed as means ± SE (n = 3)

Fresh weight exhibited a differential response to priming treatments between the Lizan and Pahleh ecotypes. The Lizan ecotype demonstrated the highest fresh weight under GA treatments, with GA 75 and 150 mg/L resulting in increases of 89.22% and 90.32%, respectively, compared to the control (Fig. 3a). A distinct pattern in dry weight variation was observed across priming treatments and ecotypes. The Lizan ecotype exhibited significantly higher dry weight under most treatments. Notably, the highest dry weight in Lizan was recorded under GA 75 mg/L, with a 187.86% increase compared to the control, while in the Pahleh ecotype, GA 600 mg/L resulted in the highest dry weight, increased almost nine times. Among H treatments, only the 24-h treatment in the Lizan ecotype resulted in a significant increase, with a 153.88% rise compared to the control (Fig. 3b). Among GA treatments, GA at 75 mg/L in Pahleh resulted in a 214.28% increase in stem length compared to the control. Additionally, KN 50 in Lizan led to a statistically significant increase of 129.54% (Fig. 3c). HW treatment (1 min) significantly increased stem length compared to the control, with a rise of 78.41% in Lizan and 67.53% in the Pahleh ecotype (Fig. 3c). GA, HW, and H treatments had no significant effect on root length; however, specific treatments, including KN, SA (regardless of UV-B application), and GA at 600–1200 mg L⁻^1^, exerted a negative impact on root length (Fig. 3d).

### Principal component analysis of germination and initial seedling growth performance of *S. striata* ecotypes

**Fig. 4:**
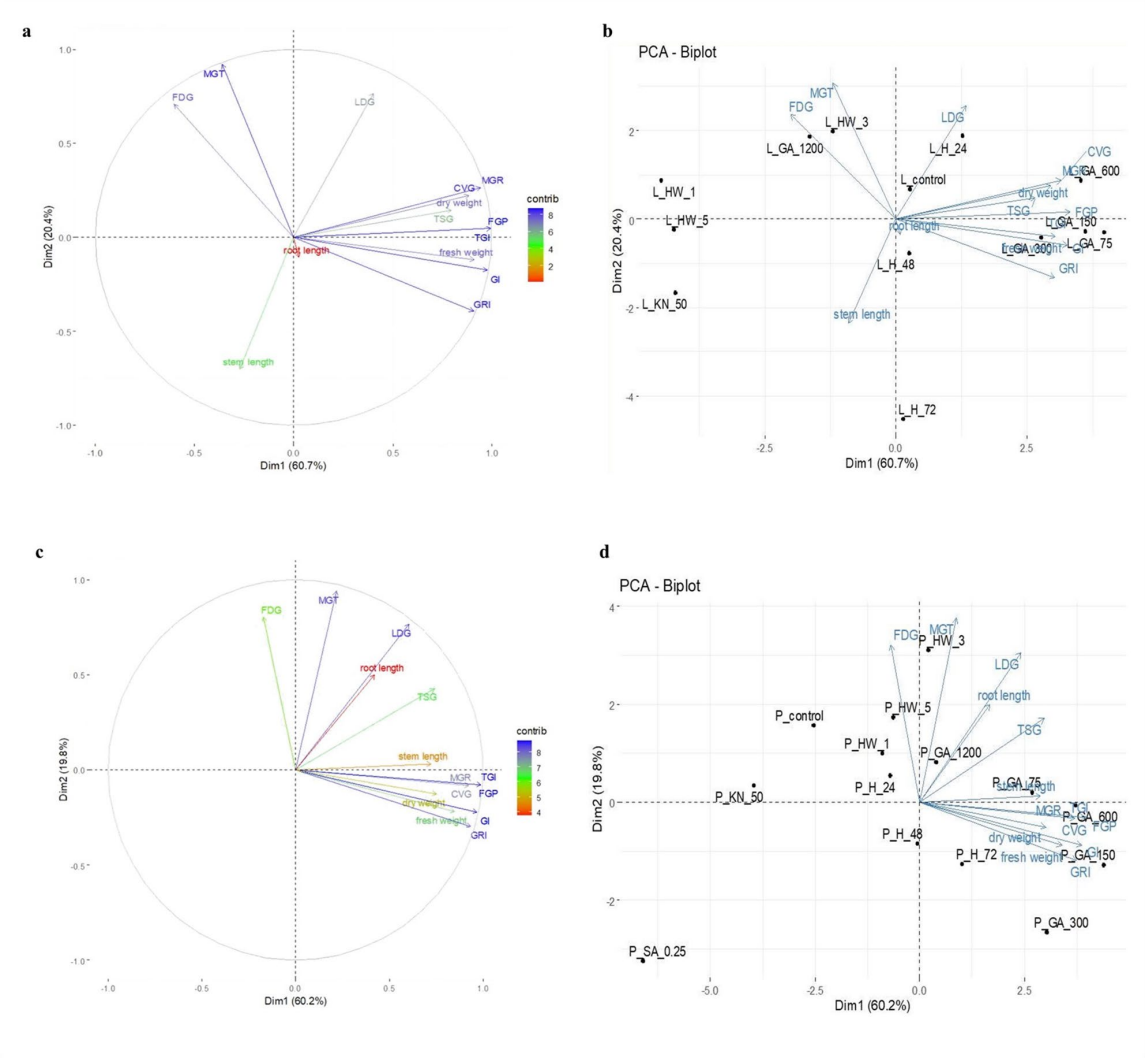
**a** PCA correlation circle of Lizan (L) ecotype germination parameters, **b** PCA biplot visualization of Lizan ecotype germination traits and experimental treatments, **c** PCA correlation circle of Pahleh (P) ecotype, and **d** PCA biplot analysis of Pahleh ecotype. first day of germination (FDG), mean germination time (MGT), mean germination rate (MGR), coefficient of the velocity of germination (CVG), final germination percentage (FGP), Timson’s germination index (TGI), germination index (GI), germination rate index (GRI)

PCA analysis of the Lizan and Pahleh ecotypes revealed that PC1 and PC2 together explained 81.1% and 80% of the total variance, with PC1 alone accounting for 60.7% and 60.2%, respectively. In Lizan (Fig. 4a, b), root length loaded strongly on PC1, while stem length dominated PC2, reflecting their distinct contributions. Fresh weight, dry weight, and final germination percentage clustered closely, indicating strong positive correlations, whereas mean germination time (MGT) and first day of germination (FDG) showed independent effects. In Pahleh (Fig. 4c, d), PC1 primarily captured variation in root length, fresh weight, dry weight, and germination indices such as final germination percentage (FGP) and Timson’s germination index (TGI), while PC2 reflected stem length, mean germination rate (MGR), and FDG. This pattern suggests that Pahleh displayed more uniform contributions across multiple germination and seedling traits compared with Lizan. Correlation circles (Fig. 4a, c) and biplots (Fig. 4b, d) consistently highlighted root length, stem length, and germination-related traits as major drivers of variance, identifying the traits most relevant to early seedling vigor and growth potential.

###  Clustering analysis of Lizan and Pahleh ecotypes

**Fig. 5:**
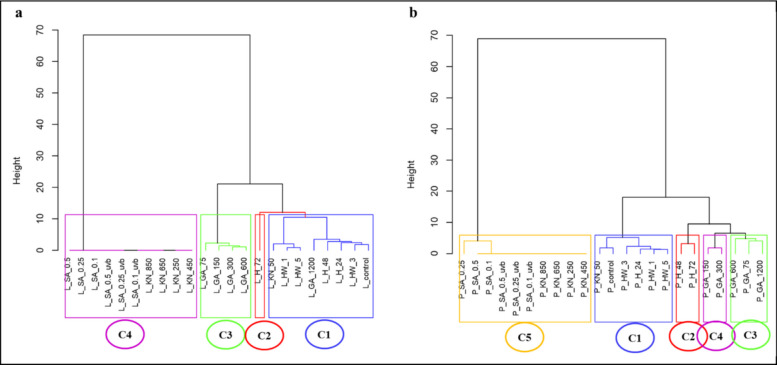
Clustering of applied treatments in **a**. Lizan and **b**. Pahleh ecotypes. H_24, 48, and 72 (24, 48, and 72 h hydropriming), GA_75, 150, 300, 600, and 1200 [gibberellin (GA) 75, 150, 300, 600, and 1200 mg/L], KN_50, 250, 450, 650 and 850 [Potassium nitrate (KN) 50, 250, 450, 650 and 850 mM], HW_1, 3, and 5 (hot water soaking for 1, 3, and 5 min), and SA_0.1, 0.25, and 0.5/_UV-B (salicylic acid 0.1, 0.25 and 0.5 mM with and without UV-B exposure)

Hierarchical clustering of the Lizan ecotype revealed four distinct clusters (C1–C4) based on the similarity of germination and seedling traits (Fig. 5a). Cluster C1 included treatments such as L_GA_1200, L_H_48, and L_control, forming a compact group with high similarity. Within C1, a smaller subgroup, L_KN_50, was closely associated with HW_1 and HW_5 but slightly separated from the main subcluster. Cluster C3 contained treatments such as L_GA_150, L_GA_300, and L_GA_600, showing moderate separation from L_GA_75 in the other subgroup. Cluster C4 included a broader range of treatments, including L_SA_0.25, L_SA_0.1, and L_KN_850, indicating greater variability within this cluster. The height of dendrogram branches reflects the degree of dissimilarity between clusters, with C1 and C4 being the most distinct. Cluster C1 was tightly grouped, potentially representing a baseline set of treatments with more consistent traits, while C2 formed a distinct subgroup, and C3 showed moderate divergence. Cluster C4 was the most heterogeneous, suggesting higher variability among the treatments. Overall, the clustering patterns reveal clear differences in treatment responses across the Lizan ecotype, which may have implications for germination performance and early seedling growth.

For the Pahleh ecotype (Fig. 5b), hierarchical clustering revealed five main clusters (C1–C5) among the priming treatments. Cluster C1 included control, low KN, short H, and short HW treatments, forming a compact group with similar germination responses. Cluster C2, consisting of longer H durations, was separated slightly from C1, reflecting the distinct effects of extended soaking on seed germination and early seedling growth. Cluster C3, representing high and low GA concentrations, showed moderate separation from other clusters, while cluster C4, containing moderate GA treatments, formed a distinct subgroup. Cluster C5 included SA treatments (with and without UV-B) and higher KN concentrations, representing the most heterogeneous responses. The branch heights indicate that C1 and C5 are the most distinct clusters, with clusters C2, C3, and C4 at intermediate distances. Overall, the clustering structure reveals clear differences in treatment responses among priming strategies in Pahleh, which may have implications for optimizing germination and early seedling growth.

### Multidimensional scaling analysis indicating the effectiveness of germination enhancement treatments

Germination enhancement was classified based on the percentage change in final germination percentage (FGP) relative to the control. Treatments resulting in > 30% increase were classified as *very good*, 20–30% increase as *good*, 0–19% increase as *fair*, and values below the control as *poor*.

**Fig. 6:**
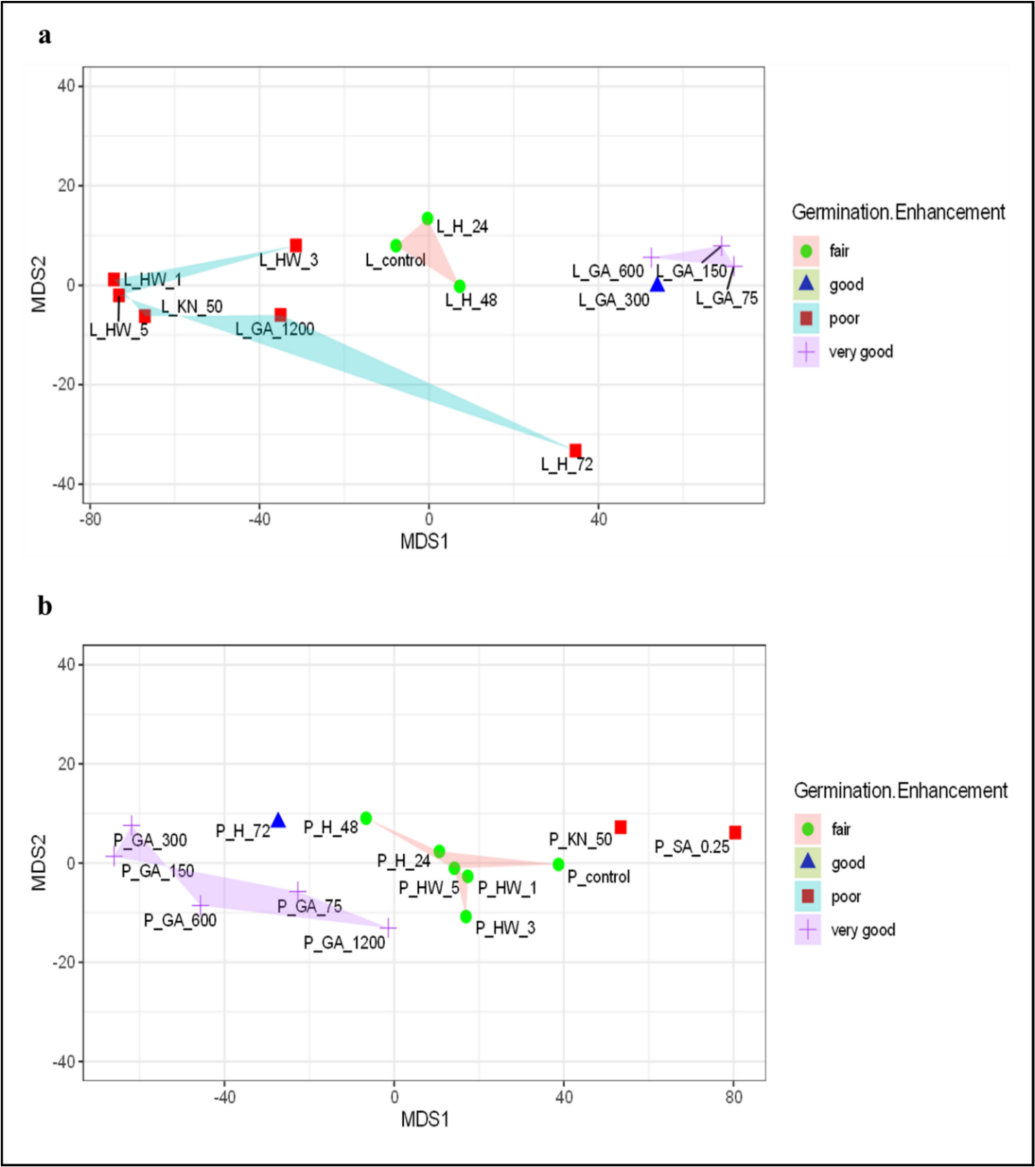
Multidimensional scaling (MDS) Plot of germination enhancement in **a**. Lizan (L prefix) and **b**. Pahleh (P prefix) ecotypes after multiple treatments. The green circles, representing "fair" germination enhancement, blue triangles represent "good" enhancement, red squares show "poor" germination enhancement, and purple crosses represent "very good" enhancement. H_24, 48, and 72 (24, 48, and 72 h hydropriming), GA_75, 150, 300, 600, and 1200 (gibberellin (GA) 75, 150, 300, 600, and 1200 mg/L), KN_50, 250, 450, 650 and 850 (Potassium nitrate (KN) 50, 250, 450, 650 and 850 mM), HW_1, 3, and 5 (hot water soaking for 1, 3, and 5 min), and SA_0.1, 0.25, and 0.5/_UV-B (salicylic acid 0.1, 0.25 and 0.5 mM with and without UV-B exposure)

The MDS plot of Lizan and Pahleh ecotypes illustrates the relationships among various seed treatments based on their effectiveness in enhancing germination. Treatments are color- and shape-coded to denote different categories of germination enhancement, as explained in the legend. In Lizan's MDS plot, the stress value of 0.0334 indicates an excellent fit of the two-dimensional projection to the original distance matrix, ensuring high confidence in the visual representation of the data (Fig. 6a). Each treatment is represented by a labeled point in the MDS space, with positions determined by the similarity or dissimilarity in germination enhancement outcomes. According to clustering patterns, treatments grouped by proximity in the MDS space reflect similar germination enhancement responses. Treatments with "poor" germination enhancement) included L_HW_1, L_HW_3, L_HW_5, L_KN_50, L_GA_1200, and L_H_72, indicating their mutual similarity in achieving suboptimal results. Moreover, "fair" germination enhancement, including L_H_24 and L_H_48 points centrally located in the plot, and clustered tightly around the L_control. The clustering of the "fair" treatments around the L_control suggests that these treatments provide a modest benefit over untreated seeds, but do not achieve the effectiveness of more targeted growth regulator interventions. The group of L_GA_300 and L_GA_600 on the right side of the plot illustrates their intermediate performance in germination improvement. L_GA_75 and L_GA_150 form a distinct group, highlighting their superior germination improvement in Lizan compared to other treatments. Even though lower concentrations of GA in Lizan ecotype (e.g., L_GA_75, L_GA_150) were associated with superior enhancement ("excellent"), high concentrations (e.g., L_GA_1200) or heat-based treatments (e.g., L_HW_1, L_HW_5) tend to perform poorly. The low-stress value of 0.0334 confirms that the two-dimensional representation is a reliable simplification of the underlying relationships among treatments. The separation of categories, the observed clustering, and the spread within each category can be used to infer treatment efficacy, consistency, and potential avenues for optimizing germination enhancement strategies (Fig. 6a). The MDS plot of the Pahleh ecotype (stress = 0.0184, indicating an excellent fit) illustrates the relationships among seed treatments based on germination enhancement effectiveness. The plot highlights distinct clustering patterns: "poor" treatments (P_KN_50, P_SA_0.25) are isolated on the right, while "excellent" treatments (P_GA_75, P_GA_150, P_GA_300, P_GA_600) cluster tightly on the left. "Fair" treatments (P_control, P_HW_1, P_HW_5, P_H_24, P_H_48) group centrally, suggesting intermediate performance. The "good" treatment (P_H_72) lies closer to the "excellent" cluster, indicating higher enhancement potential. Shaded polygons emphasize the cohesiveness of each category. These results demonstrate clear distinctions among treatments, with growth regulator-based methods (P_GA) outperforming heat treatments (P_HW) and controls. This visual provides robust insights for optimizing germination strategies. (Fig. 6b).

## Discussion

The present study was designed to evaluate whether different seed priming strategies enhance germination and early seedling performance of *S. striata*, and whether these responses are ecotype-dependent. Overall, the findings support our hypotheses that priming treatments significantly modify both the capacity (FGP) and the kinetics (FDG, MGT, MGR) of germination (Fig. 1, 2). However, the magnitude and direction of these effects vary according to the priming treatments and ecotypes, suggesting that both the type of priming agent and genetic background play critical roles in regulating germination behavior. Importantly, multivariate analyses revealed coordinated shifts among germination capacity, speed, and growth traits, highlighting structured treatment-specific response patterns that would not have been evident from single-trait analyses alone (Fig. 4–6).

GA plays an important role in seed germination by alleviating dormancy and promoting embryo growth. However, their regulatory effects are highly concentration-dependent and closely modulated through interactions with other phytohormones, particularly ABA [39]. In the present study, high GA concentration (1200 mg/L, Lizan ecotype) delayed germination and reduced FGP, suggesting that excessive GA disrupt hormonal balance and reduce germination efficiency (Fig. 1). The increase in FDG and MGT under high GA further suggests delayed metabolic activation and reduced synchrony within the seed population (Fig. 1). In contrast, moderate GA concentrations (75 and 150 mg/L) improved germination kinetics, reflected in reduced MGT and increased MGR, indicating faster and more synchronized germination (Fig. 1, 2). These kinetic improvements were accompanied by increased dry biomass accumulation in the Lizan ecotype, suggesting that optimal GA levels enhance both reserve mobilization and post-germinative growth (Fig. 3). A similar concentration-dependent pattern has been reported in dwarf schefflera, where appropriate GA levels stimulated vegetative growth, whereas excessive concentrations were inhibitory [40]. The promoting effect of moderate GA concentrations likely reflects enhanced α-amylase activity and improved starch mobilization, which provide soluble sugars required for embryo growth and rapid radicle protrusion [41–44]. However, the inhibitory effects observed at high concentrations emphasize that GA-mediated enhancement of germination is highly dose-dependent and species-specific, as contrasting biomass responses have been reported in tomato [45]. Collectively, these findings indicate that GA primarily influenced both germination capacity (FGP) and germination speed (MGT, MGR), but only within the optimal concentration window, emphasizing the importance of optimizing GA concentrations in priming treatments.

Hydropriming (H) primarily influenced germination timing rather than final capacity. In the Lizan ecotype, H (72 h) significantly reduced FDG, indicating earlier initiation of germination, while FGP remained relatively stable (Fig. 1). This suggests that hydropriming accelerated early metabolic reactivation without substantially altering overall viability. The reduction in FDG and MGT under H (Fig. 1) implies improved synchronization and faster radicle emergence, likely due to enhanced water uptake and activation of hydrolytic enzymes, such as α-amylase, which facilitates the mobilization of seed nutrient reserves [46]. Moreover, H induces biochemical changes, including increased antioxidant enzyme activity and improved metabolic function, ultimately supporting germination and subsequent seedling growth [46]. However, the limited change in FGP, especially in Lizan, indicates that water uptake alone may not be sufficient to overcome intrinsic dormancy constraints (Fig. 1). In contrast, HW soaking reduced germination rate, as reflected by increased FDG and MGT, suggesting transient thermal stress or partial damage during imbibition (Fig. 1, 2). Nevertheless, HW treatment (1 min) significantly increased stem length in both ecotypes, indicating that while early germination kinetics may have been slightly delayed, subsequent seedling growth was stimulated. Similar improvements in germination percentage and seedling vigor following HW treatments have been documented in several species. For instance, water soaking at ≃50°C for 30 min significantly improved seedling length, dry weight, and vigor indices in *Capsicum annuum* [47]. Similarly, HW pre-treatment increased cumulative germination percentage in *Falcataria falcata* [48]. HW treatment is thought to accelerate water uptake, thereby initiating germination and enhancing metabolic activity, including the accumulation of amino acids, sugars, and organic acids essential for seedling development [4]. In addition, HW can break physical dormancy, particularly in seeds with hard coatings, which may explain its positive effects in some species. However, the response to HW treatment is not always consistent and depends on species, ecotype, and exposure duration. While HW soaking improved seedling emergence and early growth in *Leucaena leucocephala*, it was less effective than acid scarification [49]. Prolonged exposure (65°C for 30–90 min) negatively affected germination in *Indigofera zollingeriana* and did not significantly influence seedling height, indicating that excessive heat can be damaging [50]. Likewise, in *Platycodon grandiflorum*, other soaking treatments such as GA and KNO3 were more effective in promoting germination and seedling growth [51]. In contrast, HW treatment at 52 °C for 30 min improved biomass in *Abelmoschus esculentus* [52]. Overall, H moderately improved germination parameters, but its effects were less pronounced than those of chemical or physical treatments, suggesting that water uptake alone may be insufficient to induce the physiological changes required for optimal germination. In addition, hydrothermal priming mainly modulates germination kinetics (FDG, MGT) and post-germinative growth rather than substantially altering FGP.

SA priming generally suppressed germination performance in both ecotypes. Reductions in FGP under most SA concentrations indicate decreased final germination capacity, suggesting impaired embryo viability or inhibition of reserve mobilization. Increases in FDG and MGT, together with reductions in MGR, further indicate delayed and less synchronized germination. This inhibitory effect may result from SA-induced enhancement of ABA signaling and suppression of GA-mediated α-amylase activity, thereby limiting starch hydrolysis and energy availability for the embryo [53, 54]. Elevated ROS levels, under high SA concentrations, may further contribute to oxidative stress during early imbibition. Consistent with the present findings, high SA concentrations inhibited all growth processes in the root of *Arabidopsis thaliana* [55], whereas lower concentrations (e.g., 0.025–0.05 mM) enhanced germination indices in species such as chamomile and *Tagetes* spp., and higher concentrations (e.g., 0.2 mM) had inhibitory effects, indicating a concentration-dependent impact [56]. The positive response observed at 0.25 mM SA in the Pahleh ecotype suggests genotype-specific sensitivity thresholds. Similar to this positive response, in another study, SA improved the length and dry weight of barley species in addition to increasing the germination rate and mean daily germination [55]. These positive effects were associated with enhanced cell division, improved nutrient mobilization, and better ionic balance during early developmental stages, likely mediated by increased H⁺-ATPase activity and improved K⁺ uptake. Additionally, SA contributes to membrane stability and antioxidant regulation, supporting vigorous seedling growth. This result emphasizes that the effect of SA treatment is strongly dependent on the plant species [55]. SA also interacts with other hormones to regulate germination, and it has been reported to stimulate GA biosynthesis under stress conditions, which may promote germination. In line with the dose-dependent inhibition observed here, SA has been reported to suppress germination by blocking GA-stimulated α-amylase production in barley, thereby preventing starch mobilization required for seedling growth [40]. Similarly, higher SA concentrations (100–500 μM) inhibited germination in *Secale cereale* L., with complete inhibition at 1000 μM, accompanied by biochemical and developmental alterations [57]. SA has also been reported to modulate GA biosynthesis under stress conditions; however, excessive SA may disrupt the ABA/GA balance necessary for coordinated germination, which may explain the suppression observed in the current study under SA treatments [58]. The variability of SA effects among species and genotypes is further supported by findings in wheat, where SA inhibited germination more strongly than ABA without a clear genotype correlation [59]. Therefore, the present results reinforce that SA predominantly affected germination capacity (FGP) and synchrony (MGT, MGR), with strong ecotype dependence. However, contrasting results between the present study and previous reports may arise from species- and genotype-specific responses, as well as differences in experimental conditions [60].

In most evaluated traits, KN application significantly reduced germination performance relative to the control. Decreases in FGP and increases in MGT indicate reduced final capacity and slower germination under higher KN concentrations (Fig. 1, 2). Similar results were observed in tomato in suppressing germination parameters when > 1% KN was applied [61], but an opposite result was observed in *Ocimum tenuiflorum* [62]*.* However, at 50 mM, stem length in the Lizan ecotype increased significantly, suggesting that moderate nitrate levels may promote post-germinative growth despite limited improvements in germination kinetics. This observation is similar to those on Amaranth [63]. The reduction in root length of both ecotypes at this concentration (50 mM KN) suggests differential allocation patterns between shoot and root tissues and a negative effect of higher KN levels on root development as observed in carrot seedlings [64]. Nitrate is known to act as a signaling molecule that reduces dormancy and promotes germination, likely through modulation of hormonal pathways [65]. This effect is often attributed to KN-mediated changes in ABA metabolism, including increased GA levels and reduced ABA concentrations, which together lower the ABA/GA ratio and enhance germination [66, 63]. Its promotive effects are often associated with lowering the ABA/GA ratio, thereby enhancing germination speed. Nevertheless, excessive KN concentrations may create osmotic or ionic stress, explaining the increased MGT and reduced FGP observed here. Similar concentration-dependent effects have been reported in tomato, carrot, and other species [62, 67, 68]. Nitrogen helps seeds to synthesize proteins, which affects the quality of seeds [62]. Although KN has been shown to enhance antioxidant enzyme activities, thereby protecting seeds from oxidative stress during germination and improve germination percentage in several species [68–70], the present results indicate that in *S. striata*, its effectiveness is strongly ecotype- and dose-dependent. Collectively, these findings support the role of nitrate as a signaling molecule that interacts with ABA and GA pathways to promote germination [65]. Thus, KN primarily modulated germination kinetics and early seedling growth rather than consistently improving final germination capacity. Overall, the results of the multivariate analyses indicated that the Lizan and Pahleh ecotypes exhibited distinct responses to germination-enhancing treatments. PCA revealed that most of the variation was explained by traits associated with initial seedling vigor, particularly root length, shoot length, biomass, and germination indices, although the relative importance of these traits differed between ecotypes. In the Lizan ecotype, trait effects were partially dissociated, whereas in the Pahleh ecotype, responses were more coordinated and integrated across germination and growth attributes. Hierarchical clustering and MDS further clearly grouped treatments according to their effectiveness, and the satisfactory model fit confirmed the robustness of the results. In both ecotypes, low to moderate concentrations of GA yielded the best performance, whereas high GA concentrations, thermal treatments, and certain KN or SA treatments generally exhibited weak or unstable effects. Collectively, these findings highlight the importance of root- and germination-related traits and underscore the need to optimize priming treatments in an ecotype-specific manner to improve early seedling establishment in *S. striata*. This experiment highlights the importance of selecting appropriate priming methods based on plant species, ecotype, and seed physiology to optimize germination and seedling performance.

## Conclusion

This research thoroughly examines different seed priming methods to improve the germination of *S. striata* under a range of priming conditions. The results provide valuable guidance for improving seed treatment strategies in medicinal plants by combining chemical, physical, and environmental priming methods. The study indicates that priming treatments influence germination, either positively or negatively, with effects varying depending on the treatment type, plant ecotype, and concentration. In addition, employing advanced statistical techniques like MDS and cluster analysis offered fresh insights into how different priming treatments influence germination responses. This approach provides a clearer and more precise understanding of seed behavior, supporting the development of agricultural practices based on solid data. With growing environmental challenges impacting seed germination, the study highlights the importance of combining seed priming strategies. Future research should delve deeper into the molecular and physiological processes behind priming-driven improvements in germination, paving the way for more targeted and efficient seed treatment techniques. Using these perspectives can improve germination rate and plant establishment, and help sustainable agriculture.

## Supplementary Information

Supplementary Material 1.

## Data Availability

The dataset generated during and/or analyzed during the current study is available from the corresponding author upon reasonable request.

## References

[1] Lamichhane JR, Debaeke P, Steinberg C, You MP, Barbetti MJ, Aubertot J-N. Abiotic and biotic factors affecting crop seed germination and seedling emergence: a conceptual framework. Plant Soil. 2018;432:1–28.

[2] Bhatla SC, Kathpalia R. Seed dormancy and germination. In: Plant physiology, development and metabolism. Springer; 2023. p. 625–40.

[3] Cantliffe DJ, Fischer JM, Nell TA. Mechanism of seed priming in circumventing thermodormancy in lettuce. Plant Physiol. 1984;75:290–4. 16663613 10.1104/pp.75.2.290 PMC1066899

[4] Jatana BS, Grover S, Ram H, Baath GS. Seed priming: molecular and physiological mechanisms underlying biotic and abiotic stress tolerance. Agron J. 2024;14:2901.

[5] Pawar VA, Laware SL. Seed priming a critical review. International Journal of Scientific Research in Biological Sciences. 2018;5:94–101.

[6] Tapfumaneyi L, Dube P, Mavengahama S, Ngezimana W. Effects of different levels of gibberellic acid and potassium nitrate solutions on the emergence and seedling vigor of amaranth and *Cleome gynandra*. Agrosyst Geosci Environ. 2024;7:e20464.

[7] Habibi N, Terada N, Sanada A, Koshio K. Alleviating salt stress in tomatoes through seed priming with polyethylene glycol and sodium chloride combination. Stresses. 2024;4:210–24.

[8] Jarrar H, El-Keblawy A, Albawab M, Ghenai C, Sheteiwy M. Seed priming as a promising technique for sustainable restoration of dryland. Restor Ecol. 2024;32:e14182.

[9] Karunakaran V, Sivakumar P, Pandiyan M, Mathiyazhagan S, Selvamurugan M, Baskaran R, et al. Effects of Plant Growth Regulators and Micronutrients on Crop Performance, Seed Germination and Seedling Vigour in Rice (ORYZA SATIVA L.). Appl Ecol Environ Res. 2024;22. 10.15666/aeer/2201_709719.

[10] Fu Y, Ma L, Li J, Hou D, Zeng B, Zhang L, et al. Factors influencing seed dormancy and germination and advances in seed priming technology. Plants. 2024;13:1319. 38794390 10.3390/plants13101319 PMC11125191

[11] Ambrico PF, Šimek M, Morano M, De Miccolis Angelini RM, Minafra A, Trotti P, et al. Reduction of microbial contamination and improvement of germination of sweet basil (Ocimum basilicum L.) seeds via surface dielectric barrier discharge. J Phys D Appl Phys. 2017;50:305401.

[12] Wang W, Wang X, Zhang J, Huang M, Cai J, Zhou Q, et al. Salicylic acid and cold priming induce late-spring freezing tolerance by maintaining cellular redox homeostasis and protecting photosynthetic apparatus in wheat. Plant Growth Regul. 2020;90:109–21.

[13] Espanany A, Fallah S, Tadayyon A. Seed priming improves seed germination and reduces oxidative stress in black cumin (*Nigella sativa*) in presence of cadmium. Ind Crops Prod. 2016;79:195–204.

[14] Thomas DTT, Challabathula D, Puthur JT. UV-B priming of Oryza sativa var. Kanchana seedlings augments its antioxidative potential and gene expression of stress-response proteins under various abiotic stresses. 3 Biotech. 2019;9:375. 10.1007/s13205-019-1903-5 PMC6765627 31588399

[15] Lema SC, Kitano J. Hormones and phenotypic plasticity: implications for the evolution of integrated adaptive phenotypes. Curr Zool. 2013;59:506–25.

[16] Afshari T. Assessment of seed dormancy in Scrophularia striata. Seed Sci Technol. 2016;44. 10.15258/SST.2016.44.1.18.

[17] Mousavi SS, Karami A, Haghighi TM, Maggi F. Two Iranian Scrophularia striata Boiss. Ecotypes under UV-B radiation: Germination and initial growth perspective. South African J Bot. 2022;148:460–8.

[18] Saeed N, Nam H, Haq MIU, Muhammad Saqib DB. A survey on multidimensional scaling. ACM Comput Surv. 2018;51:1–25.

[19] Albert CH, Thuiller W, Yoccoz NG, Douzet R, Aubert S, Lavorel S. A multi‐trait approach reveals the structure and the relative importance of intra‐vs. interspecific variability in plant traits. Funct Ecol. 2010;24:1192–201.

[20] Dzemyda G, Sabaliauskas M, Medvedev V. Geometric MDS performance for large data dimensionality reduction and visualization. Informatica. 2022;33:299–320.

[21] Boyarski A, Bronstein A. Multidimensional scaling. In: Computer Vision: A Reference Guide. Springer; 2021. p. 836–49.

[22] Ghojogh B. Data Reduction Algorithms in Machine Learning and Data Science. 2021.

[23] Karavani B, Afshari RT, Hosseini NM, Moosavi SA, Akbari H. Evaluation of Cardinal Temperatures and Thermal Time Requirement for Germination of Scrophularia Striata and Tanacetum Polycephalum (Schultz Bip. Ssp. Heterophyllum). Plant Breed Seed Sci. 2018;78:83–97.

[24] Esmaeili H, Karami A, Hadian J, Nejad Ebrahimi S, Otto LG. Genetic structure and variation in Iranian licorice (*Glycyrrhiza glabra* L.) populations based on morphological, phytochemical and simple sequence repeats markers. Ind Crops Prod. 2020;145:112140. 10.1016/j.indcrop.2020.112140.

[25] Welch GB, Delouche JC. Environmental and structural requirements for seed storage. 2021. https://scholarsjunction.msstate.edu/seedtechpapers/59?utm_source=scholarsjunction.msstate.edu%2Fseedtechpapers%2F59&utm_medium=PDF&utm_campaign=PDFCoverPages.

[26] Mousavi SS, Karami A, Movahhed Haghighi T, Tahmasebi A. Lead, copper, zinc and aluminum tolerance in contrasting ecotypes of Scrophularia striata. Acta Ecol Sin. 2022. 10.1016/j.chnaes.2022.01.005.

[27] Haghighi TM, Saharkhiz MJ, Naddaf F. Ontogenetic variability of *Vitex pseudo-negundo* essential oil and its phytotoxic activity. Sci Hortic. 2019;257:108735.

[28] Haghighi TM, Saharkhiz MJ. Phytotoxic potential of *Vitex pseudo-negundo* leaf and flower extracts and analysis of phenolic compounds. Biocatal Agric Biotechnol. 2021;34:102018. 10.1016/j.bcab.2021.102018.

[29] Al-Mudaris MA. Notes on various parameters recording the speed of seed germination. Tropenlandwirt J Agric Trop Subtrop. 1998;99:147–54.

[30] Sharma A, Devkota D, Thapa M, Bista B. Improving germination and stand establishment of kiwifruit (*Actinidia deliciosa* cv. Hayward) seed through media selection and hormonal use in Dolakha. Nepal. Trop Agrobiodiversity. 2021;2:16–21.

[31] Marques FRF, Meiado MV, Castro NMCR de, Campos ML de O, Mendes KR, Santos O de O dos, et al. GerminaQuant: a new tool for germination measurements. J Seed Sci. 2015;37:248–55.

[32] El-Katony TM, Khedr A-HA-F, Soliman NG. Nutrients alleviate the deleterious effect of salinity on germination and early seedling growth of the psammophytic grass *Elymus farctus*. Botany. 2015;93:559–71.

[33] Oyedeji S, Oluokun OC, Agboola OO, Animasaun DA, Fatoba PO. Ameliorative effects of Daniellia-and Vitellaria-derived biochars on the chemistry of oil-contaminated soils and germination indices of cowpea. Ceylon J Sci. 2018;47:247–52.

[34] Joosen RVL, Kodde J, Willems LAJ, Ligterink W, van der Plas LHW, Hilhorst HWM. GERMINATOR: a software package for high‐throughput scoring and curve fitting of *Arabidopsis* seed germination. Plant J. 2010;62:148–59. 20042024 10.1111/j.1365-313X.2009.04116.x

[35] Ni B-R, Bradford KJ. Quantitative models characterizing seed germination responses to abscisic acid and osmoticum. Plant Physiol. 1992;98:1057–68. 16668727 10.1104/pp.98.3.1057 PMC1080308

[36] Pereira RC, Monterroso C, Macías F. Phytotoxicity of hexachlorocyclohexane: effect on germination and early growth of different plant species. Chemosphere. 2010;79:326–33. 20172584 10.1016/j.chemosphere.2010.01.035

[37] Ranal MA, Santana DGde. How and why to measure the germination process? Braz J Bot. 2006;29:1–11.

[38] Karami A, Esmaeili S, Sahrkhiz MJ. Phytotoxic activity of *Tecomella undulata* (Sm.) Seem extracts on some ornamental plants. Biocatal Agric Biotechnol. 2017;9:177–82.

[39] Vishal B, Kumar PP. Regulation of seed germination and abiotic stresses by gibberellins and abscisic acid. Front Plant Sci. 2018;9:838. 29973944 10.3389/fpls.2018.00838 PMC6019495

[40] Sardoei AS, Tahmasebi M, Bovand F, Ghorbanpour M. Exogenously applied gibberellic acid and benzylamine modulate growth and chemical constituents of dwarf schefflera: a stepwise regression analysis. Sci Rep. 2024;14:7896. 38570571 10.1038/s41598-024-57985-0 PMC10991322

[41] Kaur S, Gupta AK, Kaur N. Gibberellin A3 reverses the effect of salt stress in chickpea (*Cicer arietinum* L.) seedlings by enhancing amylase activity and mobilization of starch in cotyledons. Plant Growth Regul. 1998;26:85–90.

[42] Liu F-F, Qiao X-H, Yang T, Zhao P, Zhu Z-P, Zhao J-H, et al. Nitric oxide promoted the seed germination of *Cynanchum auriculatum* under Cadmium Stress. Agronomy. 2023;14:86.

[43] Anwar S, Shafiq F, Nisa Z, Usman U, Ashraf MY, Ali N. Effect of cadmium stress on seed germination, plant growth and hydrolyzing enzymes activities in mungbean seedlings. J Seed Sci. 2021;43:e202143042.

[44] He Y, Zhu M, Li Z, Jiang S, He Z, Xu S, et al. IPA1 negatively regulates early rice seedling development by interfering with starch metabolism via the GA and WRKY pathways. Int J Mol Sci. 2021;22:6605. 34203082 10.3390/ijms22126605 PMC8234402

[45] Qin C, Zhang Z, Xi W, Shi C, Lei H, He Z, et al. Gibberellic acid and spermine induce mercury tolerance in tomato seedlings by modulating osmolyte, redox homeostasis and detoxification system. BMC Plant Biol. 2025;25:1140. 40866815 10.1186/s12870-025-06674-9 PMC12382030

[46] Choi J-Y, Ju Y-H, Nakamichi A, Cho S-W, Woo S-H, Sakagami J-I. Effect of seed hydropriming on the elongation of plumule and radicle during the germination process and changes in enzyme activity under water-deficient conditions. Plants. 2024;13:3537. 39771234 10.3390/plants13243537 PMC11679898

[47] Singh S, Malik AK, Bharat NK, Singh H, Kumar S. Effect of abiotic hot water seed treatment on capsicum (*Capsicum annuum*) growth and yield. Indian J Agric Sci. 2020;90:809–12.

[48] Paquit JC, Luceño-Tenizo AJM, Coritico FP. Effects of the Hot Water Pre-treatments and Storage Durations on the Seed Germination of Falcata (*Falcataria Falcata* (L.) Greuter & R. Rankin). Bangladesh J Bot. 2024;53:249–55.

[49] Rusdy M. Enhancement of seedling emergence and early growth of Leucaena leucocephala by hot water, mechanical and acid scarification pre-treatments. 2017. http://www.ripublication.com/ijaes17/ijaesv12n5_11.pdf.

[50] Tahing A, Semang A, Vertygo S. The effect of hot water scarification duration on germination and growth of *Indigofera zollingeriana* seeds. Jurnal Biol Trop. 2024;24:318–24.

[51] TAN L-L. Effects of different soaking treatments on seed germination and seedling growth of Platycodon grandiflorum. Zhongcaoyao. 2013;:468–72. https://pesquisa.bvsalud.org/gim/resource/enauMartinsNetoViviana/wpr-855436.

[52] Singh S, Singh H, Bharat NK. Hot water seed treatment: a review. IntechOpen; 2020.

[53] Tania SS, Rhaman MS, Rauf F, Rahaman MM, Kabir MH, Hoque MA, et al. Alleviation of salt-inhibited germination and seedling growth of kidney bean by seed priming and exogenous application of salicylic acid (SA) and hydrogen peroxide (H2O2). Seeds. 2022;1:87–98.

[54] Pokotylo I, Hodges M, Kravets V, Ruelland E. A ménage à trois: salicylic acid, growth inhibition, and immunity. Trends Plant Sci. 2022;27:460–71. 34872837 10.1016/j.tplants.2021.11.008

[55] Pasternak T, Groot EP, Kazantsev FV, Teale W, Omelyanchuk N, Kovrizhnykh V, et al. Salicylic acid affects root meristem patterning via auxin distribution in a concentration-dependent manner. Plant Physiol. 2019;180:1725–39. 31036755 10.1104/pp.19.00130 PMC6752920

[56] Afzal I, Ashraf S, Qasim M, Basra SMA, Shahid M, Hussain B. Mannitol priming induces biochemical changes and enhances germination capacity and seedling vigor in marigold (Tagetes spp.). In: V International Symposium on Seed, Transplant and Stand Establishment of Horticultural Crops 898. 2009. p. 25–9.

[57] Yanik F, Aytürk Ö, Çetinbaş-Genç A, Vardar F. Salicylic acid-induced germination, biochemical and developmental alterations in rye (*Secale cereale* L.). Acta Bot Croat. 2018;77:45–50.

[58] Liu J, Li L, Yuan F, Chen M. Exogenous salicylic acid improves the germination of *Limonium bicolor* seeds under salt stress. Plant Signal Behav. 2019;14:e1644595. 31331225 10.1080/15592324.2019.1644595 PMC6768418

[59] Zengin F. Effects of exogenous salicylic acid on growth characteristics and biochemical content of wheat seeds under arsenic stress. J Environ Biol. 2015;36:249. 26536800

[60] Kapoor D, Gautam V, Bhardwaj R. Salicylic acid contribution in plant biology against a changing environment. Nova Science Publishers; 2021.

[61] Moaaz Ali M, Javed T, Mauro RP, Shabbir R, Afzal I, Yousef AF. Effect of seed priming with potassium nitrate on the performance of tomato. Agriculture. 2020;10:498.

[62] Thongtip A, Mosaleeyanon K, Korinsak S, Toojinda T, Darwell CT, Chutimanukul P. Promotion of seed germination and early plant growth by KNO3 and light spectra in *Ocimum tenuiflorum* using a plant factory. Sci Rep. 2022;12:6995. 35488043 10.1038/s41598-022-11001-5 PMC9054764

[63] Tapfumaneyi L, Dube P, Mavengahama S, Ngezimana W. Effect of gibberellic acid and potassium nitrate seed treatments on the emergence and seedling vigor of amaranth and *Cleome gynandra*. Agrosystems, Geosciences & Environment. 2023;6:e20359.

[64] Mahmood ur Rehman M, Liu J, Nijabat A, Alsudays IM, Saleh MA, Alamer KH, et al. Seed priming with potassium nitrate alleviates the high temperature stress by modulating growth and antioxidant potential in carrot seeds and seedlings. BMC Plant Biol. 2024;24:606. 10.1186/s12870-024-05292-1 PMC11201870 38926658

[65] Duermeyer L, Khodapanahi E, Yan D, Krapp A, Rothstein SJ, Nambara E. Regulation of seed dormancy and germination by nitrate. Seed Sci Res. 2018;28:150–7.

[66] Da Rocha LG, Rau BA, Da Silva DM, De Araújo RM, Masetto TE. Potassium nitrate (kno₃) seed priming enhances soybean seed performance. Sci Rep. 2025;15:33640. 41022977 10.1038/s41598-025-11498-6 PMC12479939

[67] Mebratu A. Potassium nitrate priming effect on the germination of tomato (*Lycopersicum esculentum*. Mill) cvs.“Mersa” and “Tekeze‐1.” Int J Agron. 2022;2022:4970107.

[68] Kanatas P, Dellaportas V, Kakabouki I, Papastylianou P. Seed priming effects on germination and first growth of the medicinal plant *Achillea millefolium* L. J Phytol. 2020;12:20–3.

[69] Hernández JA, Díaz-Vivancos P, Acosta-Motos JR, Barba-Espín G. Potassium nitrate treatment is associated with modulation of seed water uptake, antioxidative metabolism and phytohormone levels of pea seedlings. Seeds. 2021;1:5–15.

[70] Patade VY, Singh N, Grover A, Bala M. Effect of pretreatments on seed germination of musk rose (Rosa moschata Herrm.). J Appl Res Med Aromat Plants. 2025;:100628. 10.1016/j.jarmap.2025.100628.

