# Supplementary material for: Multivariate analysis of ecotypic responses of Scrophularia striata to germination-enhancing treatments
Source: BMC Plant Biol. 2026 Apr 23;26:974. doi: 10.1186/s12870-026-08440-x (PMC13238103; doi:10.1186/s12870-026-08440-x)
Supplement: Supplementary file 1 — Supplementary Material 1. [file 12870_2026_8440_MOESM1_ESM.docx]

Table S1: Calculation Formulas for Seed Germination Parameters of *Scrophularia* *striata*

| **Germination Parameters** | **Symbol** | **Unit** | **Formula for Calculation** | **Description for formula** |
| --- | --- | --- | --- | --- |
| Final Germination Percentage | FGP | % | FGP= Total seeds germinated ×100 | Total seeds germinated at the end of the trial/number of initial seeds (total seeds) |
| Mean Germination Time | MGT | Day | MGT= ΣFx/ΣF | Where F is the number of seeds germinated on day x |
| Mean Germination Rate | MGR | 1/Day | MGR= CV/100 = 1/T | Where T is the mean germination time and CV is the coefficient of velocity |
| Germination Index | GI | - | GI = (13×N1) + (12×N2) +...+ (1×N13) | N₁, N₂, … represent the number of seeds germinated on the first, second, and subsequent days, respectively, while 1, 2, … denote the corresponding weights assigned to the number of seeds germinated on each day. |
| Coefficient of Velocity of Germination | CVG | - | CVG= N1+N2+...+Ni/100 x N1T1+... +NiTi | Where N is the number of seeds germinated every day, and T is the number of days from seeding corresponding to N |
| Germination Rate Index | GRI | - | GRI= G1 / 1 + G2 / 2 + …. + Gx /X | G1= Germination percentage at first day  G1= Germination percentage on the second day and so on |
| Time Spread of Germination | TSG | Day | TSG= Tg − T0 | Tg= Time for last germination  T0 = Time for first germination |
| Timson Germination Index | TGI | - | TGI= ΣG/T | Where G is the percentage of seed germinated per day, and T is the germination period |
